# How to say goodbye

**DOI:** 10.1186/2197-425X-3-S1-A656

**Published:** 2015-10-01

**Authors:** A -S Debue, N Kentish-Barnes, G Christine, A Gressent, F Iride, C Bridey, S Calvino-Gunther, S Cosserant, S Renard, C Brossard, E Pichot, B Megarbane, K Klouche, D Flattres-Duchaussoy, C Leroy, C Bigot, B Floccard, JD Chiche, Post-ICU Study Group

**Affiliations:** Hopital Cochin, Assitance Publique Hôpitaux de Paris, Paris, France; Espace Ethique APHP, Paris, France; Hopital St Louis, Paris, France; CHU Limoges, Limoges, France; CHU Rouen, Rouen, France; Hopital Nord, Marseille, France; CHU Nancy, Nancy, France; CHU Grenoble, Grenoble, France; CHU Clermont Ferrand, Clermont Ferrand, France; CHU Caen, Caen, France; CHU Orléans, Orléans, France; Saint Joseph, Paris, France; Lariboisière, Paris, France; CHU Montpellier, Montpellier, France; CHU Nantes, Nantes, France; CHU Angers, Angers, France; CHU Lyon, Lyon, France

## Introduction

Whereas many studies have been devoted to ICU admission, there are very few data on ICU discharge policies and protocols. In qualitative studies, pts and families have reported noticeable changes in the quality of care and interest in pts, as well as feelings of abandonment. Information given to pts and relatives at the time of ICU discharge may influence long-term outcomes and psychological sequels. Caregiver's perception of ICU discharge is poorly undertood.

## Objectives

We conducted a survey to evaluate the existence ICU discharge policies and caregiver's perception of ICU discharge.

## Methods

This study was performed in 19 ICUs in France (12 MICUs, 5 mixed ICUs & 2 SICUs). We designed and conducted a 42-item survey with 3 parts: i) demographic data to assess the caregivers' profile, ii) caregiver's knowledge of outcome data & possible long-term outcomes for ICU pts, iii) caregivers's knowledge of discharge procedures in their ICU. The survey was distributed to all nurses, aid-nurses, chief-nurses and physiotherapists in order to collect ≥25 replies/centre. In addition, a senior investigator in each centre filled a questionnaire to collect centre characteristics and outcome data. This abstract reports results related to knowledge of discharge procedures.

## Results

The survey was completed by 445 caregivers (32 [IQ 27-39] y.o.; 80 % F) working in 19 ICUs as nurses (65%), aid-nurses (24%), chief-nurses (4%), physiotherapists (4%) or psychologists (0.4%). Analysis of caregivers' responses revealed that admission protocols including provision of a welcome leaflet were present in 17 out of 19 ICUs. Conversely, only 3 ICUs had a written discharge protocol and only 1 ICU provided a discharge leaflet to patients. The lack of standardization of patients' discharge policies was a matter of dissatisfaction among caregivers: 65% of caregivers claim that discharge can be significantly improved in their ICU. Caregiver's complaints related to ICU discharge include lack of patient information (40%), poor anticipation of ICU discharge (84%), and lack of family information on ICU discharge timing and consequences. Caregivers reported emergency discharge as a marker of poor quality and a cause of dissatisfaction for pts, families and caregivers. Sixty-two per cent of respondents claim that pts are never informed of this possibility. Only 4 ICUs organise post-ICU clinics for discharged pts.

## Conclusions

ICU discharge policies and protocols are infrequent in French ICUs. Lack of standardization of patients' discharge policies is a matter of dissatisfaction among caregivers.
